# Preliminary application of 3.0 T magnetic resonance chemical exchange saturation transfer imaging in brain metastasis of lung cancer

**DOI:** 10.1186/s12880-019-0400-y

**Published:** 2020-01-13

**Authors:** Yonggui Yang, Xiaobo Qu, Yihui Huang, Khan Afsar, Gen Yan, Gang Guo, Shaoyin Duan

**Affiliations:** 1 Department of Radiology, The Second Affiliated Hospital of Xiamen Medical College, Xiamen, Fujian Province China; 2 Department of Electronic Science, Fujian Provincial Key Laboratory of Plasma and Magnetic Resonance, Xiamen University, Xiamen, Fujian Province China; 3 Department of Radiology, Zhongshan Hospital Xiamen University, Xiamen, Fujian Province China

**Keywords:** Magnetic resonance imaging, Chemical exchange saturation transfer, Metastases, Magnetization transfer ratio

## Abstract

**Background:**

Lung cancer brain metastases are very common and one of the common causes of treatment failure. We aimed to examine the clinical use of chemical exchange saturation transfer (CEST) technology in the evaluation of brain metastases for lung cancer diagnosis and prognosis.

**Methods:**

We included26 cases of lung cancer brain metastases, 15 cases of gliomas, and 20 cases with normal tests. The magnetization transfer ratio (MTR;3.5 ppm) image from the GRE-EPI-CEST sequence was analyzed using the ASSET technique and APT technology. The MTR values were measured in the lesion-parenchymal, edema, and non-focus regions, and the MTR image was compared with the conventional MRI. ANOVA and t-test were used for statistical analysis.

**Results:**

The lesion-parenchymal, edema, and non-focus areas in the metastatic-tumor-group were red-yellow, yellow-green, and green-blue, and the MTR values were 3.29 ± 1.14%,1.28 ± 0.36%,and 1.26 ± 0.31%, respectively. However, in the glioma-group, the corresponding areas were red, red-yellow, and green-blue, and the MTR values were 6.29 ± 1.58%, 2.87 ± 0.65%, and 1.03 ± 0.30%, respectively. The MTR values of the corresponding areas in the normal-group were 1.07 ± 0.22%,1.04 ± 0.23%, and 1.06 ± 0.24%, respectively. Traditional MR images are in black-white contrast and no metabolic information is displayed.

The MTRvalues of the three regions were significantly different among the three groups. The values were also significantly different between the parenchymal and edema areas in the metastatic-tumor-group. There were significant differences in the MTR values between the non-lesion and edema regions, but there was no significant difference between the edema and non-focus areas. In the glioma-group, there were significant differences in the MTR values between the parenchymal and edema areas, between the parenchymal and non-focus areas, and between the edema and non-focus areas.

**Conclusions:**

CEST reflects the protein metabolism; therefore, early diagnosis of brain metastases and assessment of the prognosis can be achieved using molecular imaging.

## Background

Metabolic equilibrium and acid-base balance are important components for preserving the body’s homeostasis [1]. The shifts in intracellular and extracellular metabolites and pH values in the early stages of the diseasehave received great attention. As an important branch of magnetic resonance imaging (MRI), chemical exchange saturation transfer (CEST) has the advantages of non-radiative, non-invasive, selective T_1_ and T_2_ contrast and metabolic imaging [2, 3], extending MRI from conventional anatomical imaging to live metabolic imaging, pH imaging, and other subtypes. It extends the new field of MRI molecular imaging, providing a new means of disease diagnosis, treatment, and even prevention.

Brain metastases of lung cancer are very common and serious and are also one of the common causes of treatment failure [4, 5]. Tracking metabolite changes during brain metastases is a problem, and CEST provides a new approach for solving it. We aimed to explore the clinical use of 3.0 T MRI CEST technology in the diagnosis and prognosis evaluation of lung cancer brain metastases.

## Methods

### General information

From January to July 2018, 26 patients with lung cancer brain metastases (17 males and 9 females, with a mean age 51.36 [48–73]years), 15 patients with gliomas (10 males and 5 females, with a mean age 43.20 [38–48]years), and 20 normal controls (14 males and 6 females,with a mean age 25.87 [24–29]years) were enrolled. The primary tumor of the 26 patients with brain metastases was lung cancer, including 8 cases of adenocarcinoma, 4 cases of small cell lung cancer, 11 cases of squamous cell carcinoma, and 3 cases of large cell lung cancer.

This study was reviewed and approved by the Medical Ethics Committee of Xiamen Medical College’s Second Affiliated Hospital (Approval No: 2014004). All participants provided written informed consent.

### Imaging method

Routine MRI examination (T1WI, T2WI, T2 FLAIR), enhanced analysis(T1WI + C), diffusion-weighted imaging (DWI), susceptibility-weighted imaging (SWI), arterial spin labeling (ASL), and CEST were obtained using Discovery MR750W 3.0 T(GE Medical Systems LLC, WI USA) .

The CEST imaging parameters were as follows. Oblique axis (OAx) CEST imaging: gradient recalled echo-echo planar imaging (GRE-EPI) CEST sequence using array spatial sensitivity encoding technique (ASSET), NEX = 1, TR = 2000 ms, TE = minimum, number of shots = 1, flip angle = 20°, FOV = 24 cm × 24 cm, matrix = 128 × 128, frequency = 128, phase = 128, frequency direction = R/L, slice thickness = 4 mm, spacing = 0 mm, number of slices = 1, acquisition time: 1 min 44 s.

CEST saturation parameters: Continuous saturation, Amplitude of saturtion RF =2 ut, Duration of saturtion RF =400 ms, Number of saturtion RF =4,Crusher amplitude scale =1,Extra space after crusher =0 ms.

### Inclusion and exclusion criteria

#### Inclusion criteria

All patients had clinically and pathologically confirmed findings and available MRI results and other related data.

Two high-grade imaging physicians with more than 10 years of experience in imaging diagnostics and more than 3 years of experience as attending physiciansparticipated in the inclusion process. The above data was combined with MRI, including metastatic or intracranial primary tumors, lesion location, number, size, morphology, imaging diagnosis and differential analysis of tumor signal manifestations, lesion enhancement, and edema around the tumor. The blood supply and bleeding of the tumor were also observed on SWI images. Both doctors read the films individually, and the consistent summary opinions were confirmed as true.

Patients who were reliably reported to be healthy were included in the normal group, those with lung cancer brain metastases were included in the metastatic tumor group, and those with high-grade gliomas were included in the glioma group.

#### Exclusion criteria

The following were the exclusion criteria: 1) the imaging data did not meet the diagnostic requirements; 2) lesions less than 1 cm, or 3) intra tumor bleeding on SWI.

### Data processing and analysis

CEST post-process was done using the ADW4.6 Workstation Function tool 2.0 Research version APT software. The observed 3.5 ppm magnetization transfer rate (MTR) map was used to quantify the MTR value of the lesion parenchymal region, edema area, and non-focal area, measured in percentages, compared to the plain scan, enhancement, DWI, SWI, and ASL images of the 3.0 T MRI instrument.

While drawing the area of interest, we referenced the dominant signal on the enhanced and DWI images, to avoid cysts and necrotic zones. The lesion parenchymal area was selected to be the most prominent area of the lesion or the lower ADC value of the DWI. The edema area was selected in the edema zone within 2 cm of the tumor in conjunction with T2 FLAIR and enhancement. In the normal white matter region on the contra lateral side, the non-lesion area was selected. Both areas were selected to reduce the blood supply to vascular areas and cerebrospinal fluid areas as much as possible.

The measured data were expressed as mean ± standard deviation and statistical analysis was performed using SPSS 23.0 from IBM. The MTR values of the focal parenchyma, edema, and the non-lesion areas, each for the normal, metastatic tumor, and the glioma groups, and the corresponding areas between the groups were first analyzed by one-way ANOVA with test criterion α = 0.05. When the one-way ANOVA analysis of variance was *P* < 0.05, the independent t-test experiment was compared with the mean number of samples. P < 0.05 was considered statistically significant with the test standard α = 0.05.

## Results

**Fig. 1:**
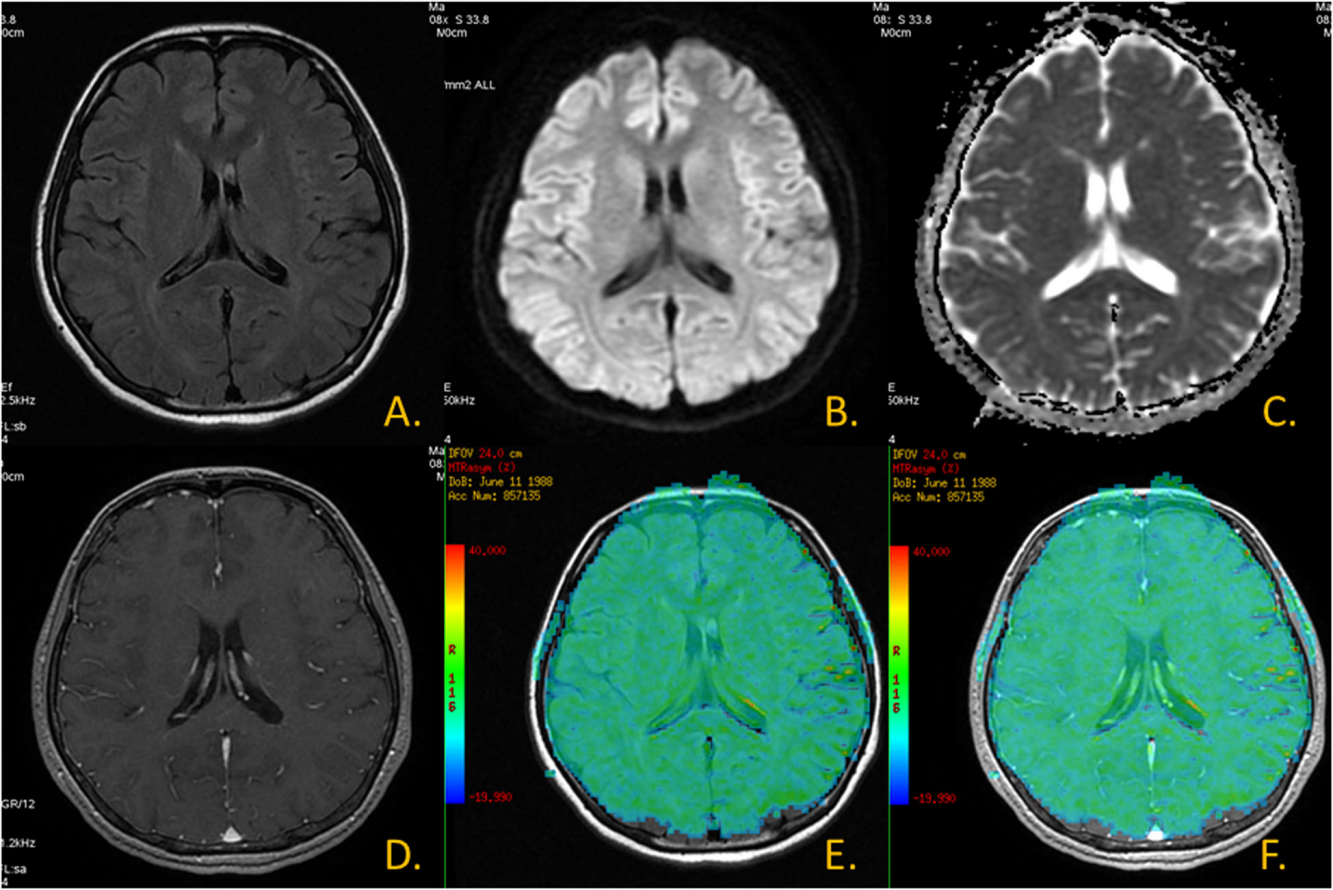
Normal volunteers. **a**.T2 FLAIR image; **b**.DWI; **c**.ADC map; **d**.T1WI + C; **e**. CEST MTR map and T2 FLAIR image fusion; **f**. CEST MTR map and T1WI + C fusion

**Fig. 2:**
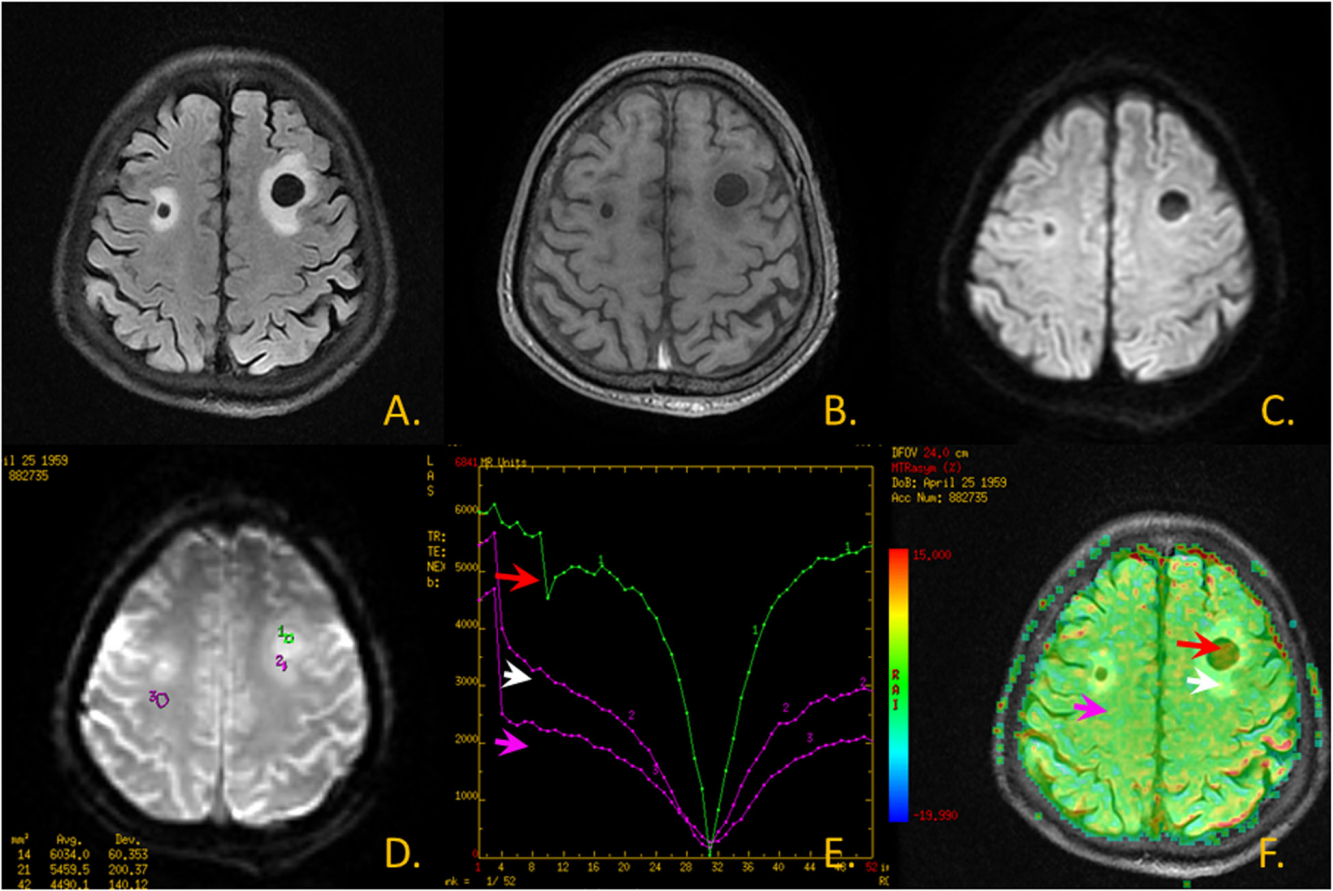
Metastatic tumor images. **a**.T2 FLAIR image; **b**.T1WI; **c**. DWI; **d**. CEST Original map; **e**. CEST Z spectrum: The red arrow area is shown as lesion-parenchymal areas, The white arrow area is shown as edema areas and The pink arrow area is shown as non-focus areas; **f**. CEST MTR map and T2 FLAIR image fusion: The red arrow area is shown as lesion-parenchymal areas, The white arrow area is shown as edema areas and The pink arrow area is shown as non-focus areas

**Fig. 3:**
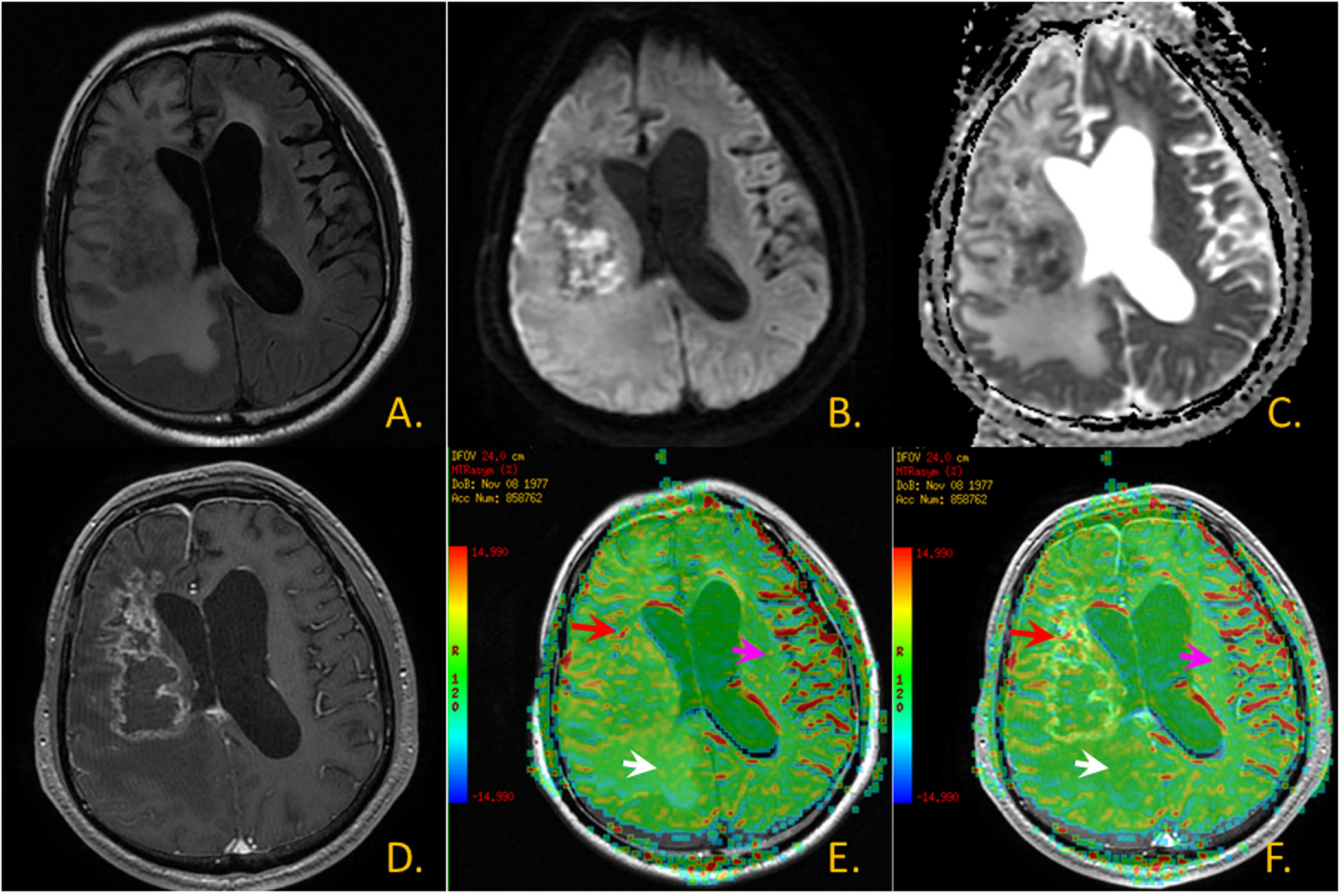
Glioma images. **a**. T2 FLAIR image; **b**. DWI; **c**. ADC map; **d**. T1WI + C; **e**. CEST MTR map and T2 FLAIR image fusion: The red arrow area is shown as lesion-parenchymal areas, The white arrow area is shown as edema areas and The pink arrow area is shown as non-focus areas; **f**. CEST MTR map and T1WI + C fusion: The red arrow area is shown as lesion-parenchymal areas, The white arrow area is shown as edema areas and The pink arrow area is shown as non-focus areas

On the MTR map, the focal parenchymal areas, the edema areas, and the non-lesion areas of the metastatic tumor group were reddish yellow, yellowish green, and greenish blue, respectively, and the MTR values ​​were 3.29 ± 1.14%, 1.28 ± 0.36%, and 1.26 ± 0.31%, respectively. These areas of the glioma group were red, reddish yellow, and greenish blue, and the boundary between the parenchyma and the edema was unclear. The corresponding MTR values were 6.29 ± 1.58%, 2.87 ± 0.65%, and 1.03 ± 0.30%. The same areas of the normal group were greenish blue, and the MTR values were 1.07 ± 0.22%, 1.04 ± 0.23%, and 1.06 ± 0.24%. Traditional MRI images are in black and white contrast with no metabolic information (Figs. 1, 2, 3, and Table 1).

**Table 1:** One-way ANOVA variance test for MTR values in corresponding groups

Groups	Number of cases	the lesion parenchymal areas	the edema areas	the non focal areas		
		MTR’s Mean ± standard deviation(%)			F value	*P* value
A	20	1.07 ± 0.22	1.04 ± 0.23	1.06 ± 0.24	0.074	0.929
B	26	3.29 ± 1.14	1.28 ± 0.36	1.26 ± 0.31	75.219	0.000
C	15	6.29 ± 1.58	2.87 ± 0.65	1.03 ± 0.30	99.167	0.000
F value		98.741	87.420	4.160		
P value		0.000	0.000	0.002		

*P* < 0.05, there is a statistical difference

A: normal group; B: metastatic tumor group; C: glioma group

The results of the one-way ANOVA analysis of variance showed that the MTR values in the focal parenchymal areas, edema areas, and the non-lesion areas of the normal group were F = 0.074, *P* = 0.929 > 0.05, no statistical difference. The MTR values in the corresponding areas of different groups and between different groups were significantly different (*P* < 0.05for all).

In the metastatic tumor and normal groups (t = − 8.550, − 2.994, − 2.403, *P* = 0.000, 0.024, 0.021) and the glioma group (t = − 6.437, − 8.399, 2.382, *P* = 0.000, 0.000, 0.024), the corresponding MTR values ​​of the corresponding three regions were statistically different (*P* < 0.05), as shown in Table 2. The MTR values ​​in the focal parenchymal areas of the metastatic tumor group were compared with those of the edema areas (t = 7.207, *P* = 0.000) and the non-lesion areas (t = 8.762, *P* = 0.000) and were all statistically different (P < 0.05). The MTR values in the edema areas and the non-lesion areas were compared, t = 0.188, *P* = 0.852, and there was no statistical difference (*P* > 0.05). The MTR values in the focal parenchymal areas of the glioma group were compared with those in the edema areas (t = 7.748, *P* = 0.000) and in the non-lesion areas (t = 12.672, *P* = 0.000), and all were statistically different (*P* < 0.05). The MTR values in the edema areas and the non-lesion areas were compared, t = 9.926, *P* = 0.000, and there was a statistical difference (*P* < 0.05), as shown in Table 3.

**Table 2:** Independent sample t-test of MTR values in corresponding regions between groups

Compared	the lesion parenchymal areas				the edema areas				the non focal areas			
	Levene’s Test		t test		Levene’s Test		t test		Levene’s Test		t test	
	F value	*P* value	t value	*P* value	F value	*P* value	t value	*P* value	F value	*P* value	t value	*P* value
normal group & metastatic tumor group	14.315	0.000	−8.550	0.000	0.597	0.445	−2.394	0.024	0.743	0.394	−2.403	0.021
normal group & glioma group	21.706	0.000	−14.642	0.000	6.538	0.015	−11.651	0.000	1.209	0.279	0.405	0.689
metastatic tumor group & glioma group	1.807	0.187	−6.437	0.000	2.959	0.095	−8.399	0.000	0.051	0.823	2.382	0.024

Levene’s Test, *P* < 0.05, homogeneity of variance; independent sample t-test, *P* = < 0.05, statistically significant

**Table 3:** Independent sample t-test of MTR values between corresponding groups in each group

Compared	metastatic tumor group				glioma group			
	Levene’s Test		t test		Levene’s Test		t test	
	F value	*P* value	t value	*P* value	F value	*P* value	t value	*P* value
lesion parenchymal areas& edema areas	10.107	0.003	7.207	0.000	7.252	0.012	7.748	0.000
lesion parenchymal areas& non focal areas	15.738	0.000	8.762	0.000	14.365	0.001	12.672	0.000
the edema areas& non focal areas	0.066	0.798	0.188	0.852	3.153	0.087	9.926	0.000

Levene’s Test, *P* < 0.05, homogeneity of variance; independent sample t-test, *P* = < 0.05, statistically significant

## Discussion

### Combination of multiple MRI techniques for brain metastases

Traditional MR diagnosis of brain metastases is performed by routine MRI and enhanced examination, and signs of brain metastases and edema around the tumor are observed. The MRI shows that the brain metastases are slightly low or low signal intensity on T_1_WI, high signal intensity on T_2_WI, equal or slightly low signal intensity on T_1_WI, and high signal intensity on T_2_WI in the edema regions around the tumor. The reason is that tumor cells have a relatively high water content than that of the normal white matter. Using MR-enhanced scans, brain metastases are distinguished using uniform enhancement, irregular enhancement, nodular or annular enhancement, and more specifically, partially hidden lesions. The edemas around the tumor are not enhanced. If intratumoral hemorrhage of the brain metastases is exacerbated by hypertension, some lesions show a high or moderately low T_1_WI signal.

The DWI and its ADC signals were used to observe the edema of lung cancer and its underlying edema. When there was a high DWI signal and decreased ADC value it reflected changes in the microscopic structure of the brain metastasis and showed other characteristics of the surrounding area of the tumor. This can effectively improve the accuracy of diagnosis and differential diagnosis of lung cancer brain metastases. This is well validated in this dataset [6].

SWI revealed tumor and peripheral blood supply, intratumoral hemorrhage, non-invasive detection of variations in magnetic sensitivity between tissues, reflecting blood oxygen levels in tissues. In this group of data, 26 cases of brain metastases and 15 cases of gliomas, SWI images showed a weak intra-tumor signal and were correlated with the blood supply artery. Cases of brain metastases and gliomas with intratumoral hemorrhage have been excluded by SWI images to avoid affecting the results of CEST MTR [7, 8].

Using ASL with PLD = 2.0, improvement in blood-brain barrier permeability and the loss of cerebral blood flow regulation can be determined during the evolution of brain metastases and gliomas [9].

### The role of 3.0 T MR CEST

In the experiment, T1WI, T2WI, T2 FLAIR, T1WI + C, DWI, SWI, and ASL of 3.0 T MRI was used for verification and observation of the metastatic tumor, glioma, and normal groups. Using the GRE-EPI-CEST sequence of ASSET technology, the 3.5 ppm (MTR) map was obtained by APT software with a quantitative analysis of the corresponding region. The CEST technique and clinical application value of 3.0 T magnetic resonance lung cancer brain metastasis were discussed. The aim was to develop a CEST-based non-invasive and accurate molecular imaging MRI research program for lung cancer brain metastases.

The results showed the characteristics of CEST signal in lung cancer brain metastasis. The focal parenchymal and edema areas of the metastatic tumor group were reddish yellow and greenish blue. The MTR value was lower than that of the glioma group, which was higher than that of the normal group. However, the non-lesion areas of the metastatic tumor group were greenish blue. The MTR value was higher than that in the normal and glioma groups. The edema and non-lesion MTR values were similar. Besides, these values were higher than those in the normal group, which were mainly in a substantial region (Figs. 1, 2 and 3) [10, 11].

This is related to the mechanism of brain metastases originating from hematogenous metastasis. Tumor cells migrate with blood in different parts of the body, including parts of the brain where no metastatic lesions have been found. As a result, the MTR value of the metastatic tumor lesions is significantly increased. The value of MTR in the edema and non-lesion areas also increased.

If the brain metastases are followed by hemorrhage, the high MTR value of the parenchymal area and the signal in the focal parenchymal areas are unequal. This is related to the content of oxygenated hemoglobin, the content of deoxyhemoglobin, and the necrosis of tumor parenchyma at different periods after hemorrhage. It can be confirmed by conventional sequences combined with SWI and ASL. When SWI showed old bleeding with a significantly low signal, the value of MTR increased by more than 80%. Although the parenchymal areas are all increased, there are wide differences between individuals and regions, which may be due to the coexistence of new and old hemorrhage in the metastatic tumors and tumor necrosis [7, 8]. CEST can detect blood products (deoxyhemoglobin, methemoglobin, ferritin, and hemosiderin) that cause hemorrhage and a significant increase in MTR. It may also be linked to changes in tissue pH during the development of metastatic brain tumors, which require further verification of the results. To avoid the interference of bleeding, the cases of intratumoral bleeding were not included in the data of this group.

PTBE will make the tumor occupying effect more apparent, further increase intracranial pressure, and exacerbate clinical symptoms. In MRI, the signal of edema around the brain metastases tumor varies. This is not only linked to macroscopic factors such as nervous system sensitivity, brain metastases position, and the degree of malignancy, but also VEGF and its receptors, AQP-4, MMP-9, IL-6, HIF-1a and other molecular factors [5, 12]. In this data, the results of VEGF and AQP-4 showed high levels of expression. Particularly in cases of peritumoral edema, AQP-4 was highly expressed in brain tissue around metastatic tumors of the brain, not in metastatic tumors. It explains why the MTR value of CEST in brain metastases is increased and the MTR value in the edema and non-focal areas is more pronounced in cases of bleeding, which may also promote angiogenesis with VEGF. This refers to the causes such as the destruction of the blood-brain barrier. The edema around the tumor is linked to the degree of malignancy of the tumor. The edema around the tumor caused by the malignant tumor which breaks the blood-brain barrier is mainly angiogenic. The edema primarily invades the white matter of the brain and the fluid with a small amount of protein is affected by the damage of the blood-brain barrier and accumulates around the tumor. The degree and magnitude of the edema around the tumor are related to the structure and characteristics of the brain tissue itself. Edema in the cortex, basal ganglia, and thalamus, for example, is not easy to develop. The edema in the white matter region is more noticeable, but not in the brain stem. In this data set, it was also confirmed that 8 cases of edema-free brain metastases occurred in the basal ganglia and thalamus.

The MTR values in the focal parenchymal areas of the glioma group were higher than that of the metastatic tumor group. In contrast, the parenchyma and edema lesion were red, reddish yellow, and their border was undefined. The non-lesion area was greenish blue, not unique from the normal non-incremental group.

The MTR values in the focal parenchymal areas of the metastatic tumor group were higher than those in the edema area and the non-focal area. The MTR values in the edema and non-lesion areas were identical, slightly higher than normal.

In this study, CEST primarily illustrated not only the metabolism of free protein and peptide molecules, but also the behavior of the tumor and its development from the molecular level [13–19]. In the edema areas of the glioma group, the MTR values are increased, consistent with the tumor surrounding the brain and the underlying edema of the tumor. Nevertheless, brain metastases do not penetrate, but compression occurs, the secondary edema of brain metastases, which varies from the initial brain tumors. CEST has certain benefits for the flexibility and precise presentation of the anatomical structure. Abundant free protein or polypeptide molecules can be visually detected in brain metastases. Furthermore, early diagnosis and thorough assessment of the extent of brain metastases and its surrounding anatomical structure [13, 18–21] are critical for its treatment and prognosis.

### Case selection principles and limitations

As a structural basis of life, proteins are closely linked to various forms of life activity. Proteins account for between 16 and 20% of body weight and their amide proton content is high [13, 18–21]. The lung cancer brain metastasis and high-grade gliomas in this research have abnormal metabolism of intracellular proteins and peptides in their disease course. This is the material basis of CEST effect in APT imaging, which is used to differentiate between parenchymal and necrotic regions, surrounding edema area, etc.

Brain metastases account for 10 to 15% of intracranial tumors and lung cancer brain metastases account for 30–40% of intracranial tumors [4]. This is also the reason why the experimental data are included in the above-described diseases.

The purpose of this experiment was to test the effect of CEST imaging. All identified brain metastases had arisen owing to lung cancer. Since the brain metastases originated from different types of lung cancer and were of different degrees, the edema and edema size were not analyzed. This, therefore, introduces the limitations of the analysis and will further refine the research in future.

At present, there are some cases of poor signal-to-noise ratio and unsatisfactory CEST image quality contrast. Combined with conventional sequence and improved examination, a certain degree of registration and correction is useful for identification and distinction of lesions, and for the study of brain metastases. The regional signal and its MTR quality can be differentiated between lesion parenchyma, necrosis, and peripheral edema (Figs. 2, 3).

The experimental group will further collect data, expand the range of disease types, randomize data, and analyze the severity and responsiveness of the disease. Simultaneously, we will perform an in-depth study of the fast-APT imaging sequences and seek to use the fast imaging sequence software package to reduce the set of interference signals generated by the EPI sequence, perform intelligent CEST-APT imaging, and provide intelligent and accurate molecular imaging diagnosis information for brain disease research.

## Conclusions

The CEST sequence and APT software using ASSET and GRE-EPI techniques obtain pseudo-color images and reflect protein metabolism. The metastatic and edema areas of the metastatic tumor group were reddish yellow and greenish blue. The level of MTR was lower than that of the glioma group, which was higher than that of the normal group. The non-focal area was greenish blue and the quality of the MTR was higher than that of the glioma and the normal groups.

Combined with the sequence of sweeping, enhancement, DWI/DKI, SWI, ASL/PWI, and MRS, the MTR image resolution and MTR value of CEST 3.5 ppm acyl protons can be used to track the distribution and metabolic changes of brain metastases and conduct early diagnosis of brain metastases and assessment of the outcome of molecular imaging of lesions.

Of course, the clinical application of CEST technology is still at the research stage and further development is required. It is assumed that the importance of its clinical application will become increasingly comprehensive with the advancement of science.

## Data Availability

All data generated or analyzed during this study are included in this published article.

## Abbreviations

APT: Amide proton transfer
AQP: Aquaporin
ASL: Arterial spin labeling
ASSET: Array spatial sensitivity encoding technique
CEST: Chemical exchange saturation transfer
DWI: Diffusion-weighted imaging
FOV: Field of view
FSE: Fast spin-echo
GRE EPI: Gradient recalled echo echoplanar imaging
HIF-1a: Hypoxia inducible factor-1a
IL: Interleukin
MMP: Matrix metalloproteinase
MRI: Magnetic resonance imaging
MTR: Magnetization transfer ratio
OAx: Oblique axis
ppm: parts per million
RF: Radiofrequency
ROI: Region of interest
SE: Spin echo
SNR: Signal to noise ratio
SWI: Susceptibility-weighted imaging
T: Tesla
T_1_WI + C: Enhanced examination (contrast) of T_1_-weighted imaging
T_1_WI: T_1_-weighted imaging
T_2_ FLAIR: T_2_fluid attenuated inversion recovery
T_2_WI: T_2−_weighted imaging
TE: Echo time
TR: Repetition time
VEGF: Vascular endothelial growth factor
